# Effect of root interaction on nodulation and nitrogen fixation ability of alfalfa in the simulated alfalfa/triticale intercropping in pots

**DOI:** 10.1038/s41598-020-61234-5

**Published:** 2020-03-06

**Authors:** Yajiao Zhao, Xiaojing Liu, Changchun Tong, Yong Wu

**Affiliations:** College of Pratacultural Science, Gansu Agricultural University, Lanzhou, China

**Keywords:** Plant sciences, Plant sciences, Systems biology, Systems biology

## Abstract

Cereal/legume intercropping is likely to achieve the optimal exploitation of soil and atmospheric nitrogen (N) sources to maintain high production and quality levels with low N inputs, as an attempt to eliminate underlying environmental effects. Nevertheless, the extent of the effect of cereal/legume intercropping on nodulation and N fixation of intercropped legumes in root interaction requires extensive verification. In the present study, root interaction of alfalfa/triticale intercropping was simulated in pots with the use of root separation types (pot with no barrier (A-T), pot with nylon mesh barrier (NA-T), pot with plastic barrier (PA-T), and alfalfa alone (SA)) in pots. Moreover, the experiment was measured at a range of N levels (N_21_, N_210_) and growing stages (branching, budding and initial flowering stages) in growth chamber. As alfalfa was growing, the total nodule number (TNN), effective nodule number (ENN) and nitrogenase activity (NA) of alfalfa with A-T and other cropping systems more noticeably differed from each other at higher N levels, whereas their diversification was reduced at lower N levels. As alfalfa was growing continuously, fresh nodule weight per plant (PNW) and single fresh nodule weight (SNW) with A-T and other cropping systems were amplified more significantly. The nodulation and N fixation ability under N_21_ were more significant than those under N_210_. Dry weight of plant per pot (TDW) and nitrogen accumulation of plant per pot (TNA) with A-T were obviously higher than those with other systems in the initial flowering stage, except for TNA under N_21_. The parameters regarding the nodulation and N fixation ability were significantly positively correlated on the whole. However, SNW and TNA were not significantly correlated, neither were SNW and TDW. According to the mentioned results, the closer root interaction, the better the nodulation form and N fixation ability of alfalfa will be, and the higher the biomass and N accumulation of all plants in pots will be. Interspecific facilitation in alfalfa/triticale intercropping system resulted in a greater yield and N accumulation; it also ultimately enhanced nodulation and N fixation ability, which can be applied in sustainable systems to avoid N loss to the environment and enhance N use efficiency.

## Introduction

The continuous input of nitrogen (N) fertilizer primarily impacts the crop production rise^1^. Nevertheless, as N fertilizer is excessively applied, low N use efficiency of crops, waste of N resources, and pollution of soil environment will be caused. Accordingly, the over application of N fertilizer does not comply with the sustainable production of agriculture^2^. A feasible cropping system and a reasonable N fertilizer application are urgently required to satisfy the needs for high yield and environmental-friendly agriculture. At present, a growing number of researchers are highlighting the cereal/legume intercropping in the sustainable agricultural development^3^. Cereal/legume intercropping refers to the way in which legume and cereal crops are planted in branches. Cereal/legume intercropping systems can largely facilitate N_2_ fixation in legumes^4,5^ and improve N exploitation in plant via synergetic mechanism of sharing soil nutrients^6^, water^7^ and nutrient uptake by plants^8^. In the meantime, cereal/legume intercropping will maintain the high production and quality levels with low N fertilizer input to eliminate underlying environmental impacts by optimally exploiting soil and atmospheric N sources, which can positively impact the intensive agricultural system^9^. Moreover, intercropped legumes are capable of transferring part of symbiotic fixed N to intercropped cereals^10^, thereby stimulating the N_2_ fixation activity of legumes^11^.

Numerous researchers reported that cereal/legume intercropping can enhance N fixation ability of legume for the strong competition of intercropped cereal for soil mineral N, as well as the increased dependence on symbiotic N_2_ fixation in cereal/legume intercropping system^8,12–14^. In this regard, in cereal/legume intercropping system, both crops exploit soil inorganic N, while legumes fix atmospheric N_2_ to provide considerable N required for optimal growth^4^. The N content in the soil is down-regulated, would induce inducing the diffusion of O_2_ in the nodule cortex, up-regulating the respiration rate of nodule and enhancing the nitrogenase activity^15^. Moreover, cereal/legume intercropping can induce the roots of cereal to secrete flavonoids to form nodules^16^. Banik and Sharma^17^ reported that intercropping of baby maize and legumes enhanced the ability of legume nodulation. Hu *et al*.^18^ considered that the nodule biomass and nodule/root biomass of intercropping pea were larger than those of the monoculture pea. Li *et al*.^19^ identified that the nodule weight and N_2_ fixation of faba bean in intercropping were higher than those of sole throughout the growing stages. In intercropping of cotton (*Gossypium hirsutum* L.) and cowpea (*Vigna unguiculata* L.), the N fixation ability of intercropping cowpea was enhanced, as compared with that of sole cropping cowpea^20^. Another example reveals that N fixation of faba bean (*Vicia faba* L.) interacting with a non-legume exerted remarkable effects compared with sole cropping legumes^12^. The mentioned studies were largely on grain crops, whereas rare studies have been conducted on forage crops. The intercropping with seed as the harvest target was ascertained in the mature period, while the intercropping with grass as the harvest target was measured before and after the initial flowering period. Thus, the root development, root exudates accumulation and nutrient competitiveness between grain crops and forage crops displayed obvious differences, and the effects on nodulation and N fixation were also different.

To satisfy the rising demand for forages, to reduce the application of N fertilizer, and to facilitate the land use, intercropping with suitable legume and cereal forage can be as adopted an effective method. Alfalfa (*Medicago sativa* L.) refers to one of the most extensively cultivated forages in arid and semi-arid areas. It is also a crucial forage crop with the merits of high yield, good palatability, high digestibility, as well as high nutritional value. Besides, alfalfa can reduce the application of N fertilizer and promote N fertilizer to be integrated in the agricultural system^21^. Triticale (*Triticale Wittmack* L.) is the cold and salt tolerant forage, characterized by high productivity and strong adaptability^22^. Moreover, triticale is a prominent ruminant feed for its high protein, critical amino acid (lysine) and starch content. At present, triticale has been increasingly accepted by the public, and its planting area is also expanding. Alfalfa/triticale intercropping can not only fully exploit light, heat, water and carbon dioxide, but also use the root nodules of alfalfa to fix N for alfalfa and triticale application. However, the nodule characteristics and biological N fixation ability and their effects on N accumulation in alfalfa/triticale intercropping remain unclear. Thus, this study hypothesizes that intercropping of alfalfa and triticale has a certain effect on the nodulation and N fixation of alfalfa. To avoid the effect of the mentioned ground interaction, and to elucidate the role of root interaction on the nodulation and N fixation of alfalfa in the intercropping, the root separation method was employed to simulate the root interaction relationship between alfalfa and triticale, and to delve into the N utilization and N transfer mechanism of intercropping.

## Results

### Nodule numbers of alfalfa

There was the extremely significant effect of N treatment in affecting TNN, ENN and ENN/TNN (*P* < 0.01) (Table 1). The effect of cropping system of TNN and ENN was extremely significant (*P* < 0.01). The TNN and ENN of alfalfa with A-T were significantly higher than that with PA-T and SA at branching and budding stages (*P* < 0.05). ENN with A-T was significantly higher than that with NA-T except that under N_21_ at branching stage (*P* < 0.05). At the same time, ENN with NA-T was significantly higher than that with PA-T and SA at initial flowering stage. No difference in ENN/TNN was witnessed in four cropping systems at branching stag. At the initial flowering stage, ENN/TNN with A-T was significantly higher than that with PA-T and SA.

**Table 1:** The total nodule number (TNN), effective nodule number (ENN), the ratio of effective nodule number/total nodule number (ENN/TNN) of alfalfa with no barrier (A-T), nylon mesh barrier (NA-T), plastic barrier (PA-T) and sole alfalfa (SA), at branching stage, budding stage, and initial flowering stage of alfalfa under N_210_ and N_21_.

N treatment	Cropping system	Branching			Budding			Initial flowering		
		TNN	ENN	ENN/TNN	TNN	ENN	ENN/TNN	TNN	ENN	ENN/TNN
N_210_	SA	12.33 ± 0.67^b^	2.44 ± 0.33^b^	0.20 ± 0.01^a^	14.00 ± 1.15^b^	3.47 ± 0.33^b^	0.25 ± 0.01^ab^	19.00 ± 1.00^bc^	7.80 ± 0.33^c^	0.41 ± 0.01^b^
	A-T	14.67 ± 0.67^a^	3.67 ± 0.67^a^	0.25 ± 0.03^a^	18.00 ± 1.00^a^	6.00 ± 0.58^a^	0.33 ± 0.02^a^	25.00 ± 0.58^a^	13.33 ± 0.67^a^	0.53 ± 0.02^a^
	NA-T	14.00 ± 0.58^ab^	2.67 ± 0.33^b^	0.19 ± 0.02^a^	17.00 ± 1.00^ab^	4.67 ± 0.33^b^	0.27 ± 0.03^ab^	22.00 ± 0.58^ab^	9.67 ± 0.67^b^	0.44 ± 0.03^b^
	PA-T	12.33 ± 0.33^b^	2.33 ± 0.33^b^	0.19 ± 0.03^a^	14.00 ± 0.58^b^	3.33 ± 0.33^b^	0.24 ± 0.04^b^	18.67 ± 1.45^c^	7.33 ± 0.33^c^	0.39 ± 0.02^b^
N_21_	SA	14.00 ± 0.58^b^	4.43 ± 0.33^b^	0.32 ± 0.01^a^	16.33 ± 0.88^b^	5.44 ± 0.33^c^	0.33 ± 0^b^	22.67 ± 0.88^ab^	10.42 ± 0.33^c^	0.46 ± 0.01^b^
	A-T	17.33 ± 0.88^a^	7.00 ± 0.58^a^	0.41 ± 0.05^a^	22.00 ± 1.15^a^	10.67 ± 0.88^a^	0.48 ± 0.02^a^	25.33 ± 0.88^a^	17.33 ± 0.88^a^	0.68 ± 0.06^a^
	NA-T	16.00 ± 1.00^ab^	5.67 ± 0.67^ab^	0.35 ± 0.03^a^	19.00 ± 0.58^ab^	8.00 ± 1^b^	0.42 ± 0.06^ab^	22.33 ± 1.20^ab^	14.33 ± 0.33^b^	0.64 ± 0.05^a^
	PA-T	13.67 ± 0.88^b^	4.67 ± 0.33^b^	0.34 ± 0.01^a^	15.67 ± 1.20^b^	5.59 ± 0.33^c^	0.36 ± 0.04^ab^	21.00 ± 1.00^b^	10.00 ± 0.58^c^	0.48 ± 0.01^b^
LSD (0.05)		2.235	1.569	0.095	2.750	2.054	0.117	3.248	1.875	0.082
Significance (*p* value)										
N treatment (N)		0.002^**^	0.000^**^	0.000^**^	0.002^**^	0.000^**^	0.000^**^	0.029^**^	0.000^**^	0.000^**^
Cropping system (C)		0.002^**^	0.002^**^	0.081	0.000^**^	0.000^**^	0.009^*^	0.000^**^	0.000^**^	0.000^**^
N × C		0.820	0.495	0.810	0.644	0.134	0.9759	0.287	0.221	0.096

Note: LSD means with different letters in the same column are significantly different at *P* < 0.05. N_21_ and N_210_ represent 21 mg L^−1^ and 210 mg L^−1^ nitrogen treatment. Branching, budding and initial flowering stage were 60, 75, and 90 days after seedling emergence of alfalfa, respectively. Here, ^*^ and ^**^ represent significance at 0.05 and 0.01 levels.

### Nodule biomass of alfalfa

The effect of N treatment of PNW and SNW was extremely significant (*P* < 0.01) (Table 2). Cropping system of PNW was extremely significant (*P* < 0.01). PNW with A-T was significantly higher than that with PA-T and SA (*P* < 0.05). In initial flowering stage, PNW with A-T was significantly higher than that with NA-T, and PNW with NA-T was significantly higher than that with PA-T and SA (*P* < 0.05). Under N_210_, SNW with A-T was significantly higher than that with PA-T (*P* < 0.05). SNW in four cropping systems had no significant difference under N_21_.

**Table 2:** Changes of two N treatment systems on fresh nodule weight per plant (PNW) and single nodule weight (SNW) of alfalfa with no root-barrier (A-T), nylon mesh barrier (NA-T), plastic barrier (PA-T) and sole alfalfa (SA), at branching stage, budding stage, and initial flowering stage.

N treatment	Cropping system	Branching		Budding		Initial flowering	
		PNW (mg plant^−1^)	SNW (mg)	PNW (mg plant^−1^)	SNW (mg)	PNW (mg plant^−1^)	SNW (mg)
N_210_	SA	1.65 ± 0.10^bc^	0.13 ± 0.01^ab^	2.45 ± 0.10^b^	0.18 ± 0.01^bc^	3.67 ± 0.11^c^	0.19 ± 0.01^ab^
	A-T	2.55 ± 0.14^a^	0.17 ± 0.00^a^	3.61 ± 0.19^a^	0.20 ± 0.00^a^	5.39 ± 0.20^a^	0.22 ± 0.01^a^
	NA-T	1.99 ± 0.18^b^	0.14 ± 0.01^ab^	3.18 ± 0.08^a^	0.19 ± 0.01^ab^	4.50 ± 0.19^b^	0.20 ± 0.00^ab^
	SA-T	1.29 ± 0.09^c^	0.10 ± 0.01^b^	2.22 ± 0.20^b^	0.16 ± 0.01^c^	3.47 ± 0.12^c^	0.19 ± 0.01^b^
N_21_	SA	4.16 ± 0.09^bc^	0.30 ± 0.02^a^	5.37 ± 0.16^c^	0.33 ± 0.01^a^	7.89 ± 0.21^b^	0.35 ± 0.01^a^
	A-T	5.60 ± 0.17^a^	0.32 ± 0.01^a^	7.65 ± 0.10^a^	0.35 ± 0.02^a^	9.25 ± 0.22^a^	0.37 ± 0.00^a^
	NA-T	5.08 ± 0.52^ab^	0.32 ± 0.02^a^	6.39 ± 0.25^b^	0.34 ± 0.01^a^	7.93 ± 0.29^b^	0.36 ± 0.03^a^
	SA-T	3.85 ± 0.33^c^	0.28 ± 0.03^a^	4.91 ± 0.15^c^	0.31 ± 0.03^a^	7.19 ± 0.38^b^	0.34 ± 0.02^a^
LSD (0.05)		0.86	0.57	0.80	0.06	0.05	0.06
Significance (*p* value)							
N treatment (N)		0.000^**^	0.000^**^	0.000^**^	0.000^**^	0.000^**^	0.000^**^
Cropping system (C)		0.000^**^	0.030^**^	0.009^**^	0.111	0.000^**^	0.434
N × C		0.531	0.806	0.06	0.982	0.424	0.997

Note: LSD means with different letters in the same column are significantly different at *P* < 0.05. N_21_ and N_210_ represent 21 mg L^−1^ and 210 mg L^−1^ nitrogen. Branching, budding and initial flowering stage were 60, 75, and 90 days after seedling emergence of alfalfa, respectively. Here, ^*^ and ^**^ represent significance at 0.05 and 0.01 levels.

### Nitrogen fixation

N treatment and cropping system had the extremely significant effect on NA and PNF (*P* < 0.01) (Table 3). At branching stage, NA with A-T was significantly higher than that with PA-T (*P* < 0.05). At budding and initial flowering stages, NA with A-T was significantly higher than that with NA-T and SA, and NA with NA-T was significantly higher than that with PA-T (*P* < 0.05). PNF with A-T was significantly higher than NA-T (*P* < 0.05), which changed from 17% to 38%. PNF with NA-T was significantly higher than that with PA-T and SA (*P* < 0.05) at budding and initial flowering stages, which changed from 36% to 61% and from 8% to 34%.

**Table 3:** Changes of two N treatment systems on nitrogenase activity of nodules (NA) and nitrogen fixation capacity per alfalfa plant (PNF) with no root-barrier (A-T), nylon mesh barrier (NA-T), plastic barrier (PA-T) and sole alfalfa (SA), at the branching stage, budding stage, and initial flowering stage.

N treatment	Cropping system	Branching		Budding		Initial flowering	
		NA	PNF	NA	PNF	NA	PNF
N_210_	SA	8.55 ± 0.061^a^	0.01 ± 0.001^bc^	8.62 ± 0.087^bc^	0.03 ± 0.001^c^	13.04 ± 0.316^b^	0.05 ± 0.003^c^
	A-T	8.91 ± 0.087^a^	0.02 ± 0.001^a^	8.91 ± 0.169^a^	0.04 ± 0.002^a^	15.54 ± 0.200^a^	0.08 ± 0.002^a^
	NA-T	8.68 ± 0.089^a^	0.02 ± 0.002^b^	8.68 ± 0.412^b^	0.03 ± 0.001^b^	13.54 ± 0.152^b^	0.06 ± 0.003^b^
	PA-T	8.26 ± 0.130^b^	0.01 ± 0.001^c^	8.26 ± 0.260^c^	0.02 ± 0.002^c^	11.45 ± 0.223^c^	0.04 ± 0.001^d^
N_21_	SA	9.94 ± 0.217^ab^	0.04 ± 0.002^bc^	11.56 ± 0.098^b^	0.06 ± 0.002^c^	15.32 ± 0.223^c^	0.12 ± 0.003^b^
	A-T	10.15 ± 0.184^a^	0.06 ± 0.001^a^	13.62 ± 0.086^a^	0.10 ± 0.002^a^	17.75 ± 0.289^a^	0.16 ± 0.006^a^
	NA-T	9.57 ± 0.382^ab^	0.05 ± 0.003^b^	11.78 ± 0.359^b^	0.08 ± 0.005^b^	16.52 ± 0.453^b^	0.13 ± 0.008^b^
	PA-T	9.28 ± 0.091^b^	0.04 ± 0.003^c^	10.15 ± 0.113^c^	0.05 ± 0.002^d^	13.17 ± 0.056^d^	0.09 ± 0.005^c^
LSD (0.05)		0.64	0.01	0.81	0.01	0.91	0.02
Significance (*p* value)							
N treatment (N)		0.000^**^	0.000^**^	0.000^**^	0.000^**^	0.000^**^	0.000^**^
Cropping system (C)		0.000^**^	0.000^**^	0.000^**^	0.000^**^	0.000^**^	0.000^**^
C × N		0.157	0.645	0.095	0.004	0.071	0.162

Note: LSD means with different letters in the same column are significantly different at *P* < 0.05. N_21_ and N_210_ represent 21 mg L^−1^ and 210 mg L^-1^ nitrogen. Branching, budding and initial flowering stage were 60, 75, and 90 days after seedling emergence of alfalfa, respectively. Here, ^*^ and ^**^ represent significance at 0.05 and 0.01 levels.

### Plant dry weight

**Figure 1:**
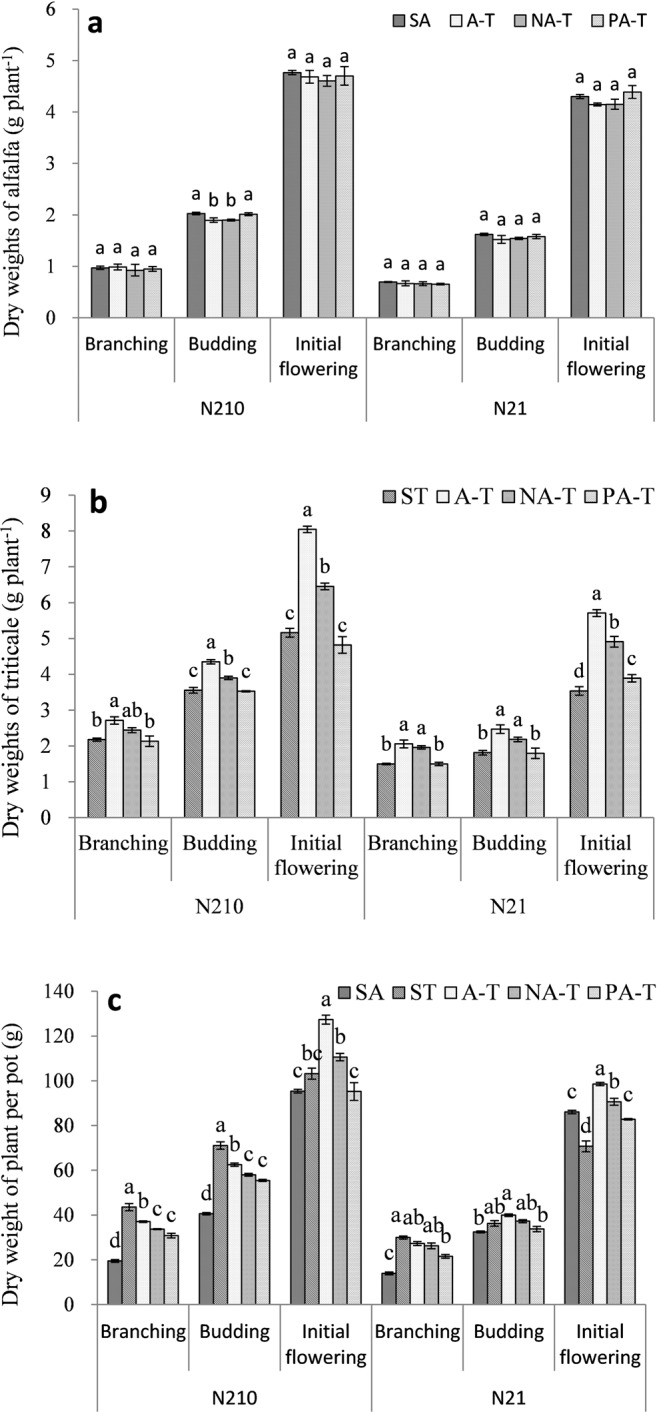
Dry weight of alfalfa (PNA) with no root-barrier (A-T), nylon mesh barrier (NA-T), plastic barrier (PA-T), sole alfalfa (SA) **(a)**; dry weight of triticale (PNA) with A-T, NA-T, PA-T, sole triticale (ST) **(b)**; dry weight of plant per pot (TNA) with A-T, NA-T, PA-T, SA and ST **(c)** at branching stage, budding stage, and initial flowering stage in N_210_ and N_21_. N_21_ and N_210_ represent 21 mg L^−1^ and 210 mg L^−1^ nitrogen. Branching, budding and initial flowering stage were, respectively, at 60, 75, and 90 days after seedling emergence of alfalfa.

There was no significant difference in PDW of alfalfa with SA, A-T, NA-T, and PA-T at three stages, except that under N_210_ at budding stage (Fig. 1a). For triticale, PDW with A-T was significantly higher than that with PA-T and ST (*P* < 0.05) (Fig. 1b**)**. At initial flowering stage, PDW of triticale with NA-T was significantly higher than that with PA-T and ST (*P* < 0.05). TDW with ST was significantly higher than that with A-T; TDW with A-T was significantly higher than that with NA-T and PA-T; TDW with NA-T and PA-T was significantly higher than that with SA under N_210_ at branching and budding stage (*P* < 0.05) (Fig. 1c). TDW with ST, A-T and NA-T was significantly higher than that with SA under N_21_ at branching stage (*P* < 0.05). At initial flowering stage, TDW with A-T was significantly higher than that with NA-T, and TDW with NA-T was significantly higher than that with PA-T and SA (*P* < 0.05).

### Nitrogen accumulate

**Figure 2:**
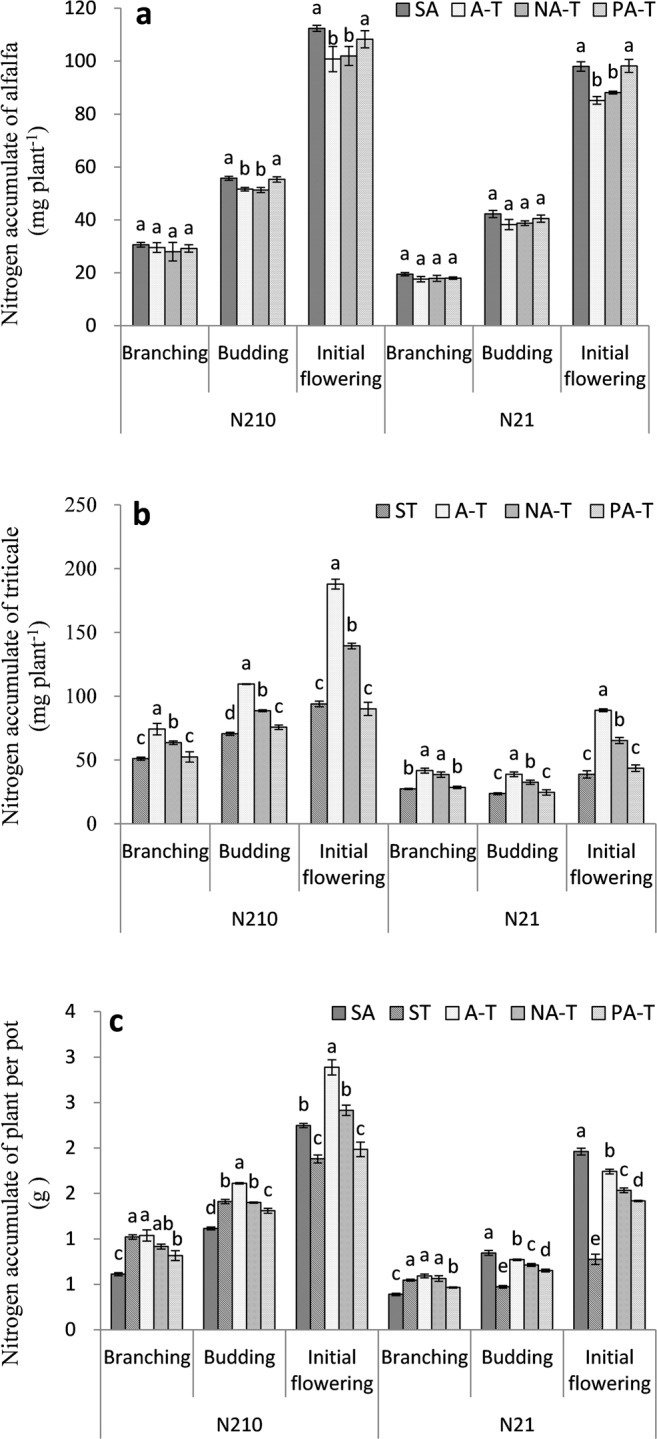
Nitrogen accumulate of alfalfa (PNA) with no root-barrier (A-T), nylon mesh barrier (NA-T), plastic barrier (PA-T), sole alfalfa (SA) **(a)**; nitrogen accumulate of triticale (PNA) with A-T, NA-T, PA-T, sole triticale (ST) **(b)**; nitrogen accumulate of plant per pot (TNA) with A-T, NA-T, PA-T, SA and ST **(c)** at branching stage, budding stage, and initial flowering stage in N_210_ and N_21_. N_21_ and N_210_ represent 21 mg L^−1^ and 210 mg L^−1^ nitrogen. Branching, budding and initial flowering stage were, respectively, at 60, 75, and 90 days after seedling emergence of alfalfa.

At budding stage under N_210_ and initial flowering stage under N_210_ and N_21_, PNA of alfalfa with SA and PA-T was significantly higher than that with A-T and NA-T (*P* < 0.05) (Fig. 2a). For triticale, PNA with A-T was significantly higher than that with NA-T; PNA with NA-T was significantly higher than that with PA-T and ST (*P* < 0.05) except it under N_21_ at initial flowering stage (Fig. 2b). Under N_210_ and N_21_, TNA with ST and A-T was significantly higher than that with PA-T, and TNA with PA-T was significantly higher than that with SA (*P* < 0.05) at branching stage (Fig. 2c). Under N_210_, TNA with A-T was significantly higher than that with NA-T, TNA with NA-T was significantly higher than that with PA-T at budding stage and initial flowering stage (*P* < 0.05). Under N_21_, TNA with SA was significantly higher than that with A-T; TNA with A-T was significantly higher than that with NA-T; TNA with NA-T was significantly higher than that with PA-T; TNA with PA-T was significantly higher than that with ST at budding and initial flowering stages (*P* < 0.05).

### Correlation analysis of different parameters

SNW and PNA-T had extremely significant negative correlation (*P* < 0.01) (Table 4). And other parameters were extremely significant positive correlation (*P* < 0.01). The correlation coefficient between PDW-A and PNA-A was the highest (0.993). The correlation coefficient between ENN/TNN and ENN was next (0.964). The correlation coefficient between PNW and PNF, ENN and NA were flowed closely (0.95). SNW and PNA-A (0.052), PNF and PNA-T (0.053) had lowest correlation.

**Table 4:** Correlation analysis of total nodule number (TNN), effective nodules (ENN), the ratio of effective nodule number /total nodule number (ENN/TNN), fresh nodule weight per plant (PNW), single nodule weight (SNW), nitrogenase activity (NA), nitrogen fixation capacity of nodules per single plant (PNF), dry weights of alfalfa (PDW-A), dry weights of triticale (PDW-T), nitrogen accumulate of alfalfa (PNA-A) and nitrogen accumulate of alfalfa (PNA-T).

	TNN	ENN	ENNTNN	PNW	SNW	NA	PNF	PDW-A	PDW-T	PNA-A	PNA-T	TDW	TNA
TNN	1												
ENN	0.915^**^	1											
ENNTNN	0.811^**^	0.964^**^	1										
PNW	0.788^**^	0.854^**^	0.859^**^	1									
SNW	0.462^**^	0.594^**^	0.691^**^	0.893^**^	1								
NA	0.903^**^	0.939^**^	0.882^**^	0.803^**^	0.526^**^	1							
PNF	0.842^**^	0.924^**^	0.895^**^	0.960^**^	0.767^**^	0.911^**^	1						
PDW-A	0.743^**^	0.700^**^	0.633^**^	0.431^**^	0.126	0.785^**^	0.560^**^	1					
PDW-T	0.691^**^	0.633^**^	0.526^**^	0.231^*^	−0.117	0.702^**^	0.401^**^	0.848^**^	1				
PNA-A	0.687^**^	0.626^**^	0.559^**^	0.354^**^	0.052	0.734^**^	0.486^**^	0.993^**^	0.838^**^	1			
PNA-T	0.434^**^	0.336^**^	0.217^*^	−0.109	−0.410^**^	0.390^**^	0.053	0.578^**^	0.903^**^	0.586^**^	1		
TDW	0.583**	0.548**	0.466**	0.262*	−0.010	0.649**	0.405**	0.758**	0.773**	0.753**	0.653**	1	
TNA	0.456**	0.404**	0.322**	0.072	−0.176	0.495**	0.212*	0.646**	0.767**	0.653**	0.739**	0.939**	1

Here, * and ** represent significance at the 0.05 and 0.01 (two tailed) level.

## Discussions

Cereal/legume intercropping is referred as to an effective planting mode. Numerous existing researches on cereal/legume intercropping are primarily focusing on food crops^18^, whereas rare studies have focused on forage crops. Alfalfa and triticale are all high-quality forages, yet the reports on intercropping between them have been rarely made. There have been considerable studies on nodules of legumes^18,23,24^, whereas the studies on nodules in the intercropping system were relatively fewer. Therefore, different root separations were adopted in this paper to study different compactness of root interaction. Moreover, the nodulation and N fixation ability of alfalfa were explored in simulated alfalfa/triticale intercropping.

### Root interaction promotes nodulation and N fixation ability

The symbiotic relationship between legumes and soil bacteria can lead to the formation of N-fixing nodules. The ability of nodulation and N fixation of legumes will be determined by the concentration of N in root environment and root exudates. The formation of nodules and the ability of N fixation of legumes can be affected by the N concentration in their rhizosphere environment and root exudates. Interspecific competitiveness and depletion of N in cereal/legume intercropping system can mitigate legume ‘N suppression’ effects and facilitate N fixation^10,25^. In the present study, compared with NA-T, PA-T and SA, A-T increased TNN, ENN ENN/TNN, PNW, SNW, NA and PNF by 5~40%, 21~96%, 7~49%, 10~55%, 2~66%, 3~35%, 8~75%, respectively. So, the nodulation (e.g., TNN, ENN, ENN/TNN, PNW and SNW) and N fixation ability (e.g., NA and PNF) with A-T were better than those with NA-T; the nodulation and N fixation ability with NA-T were more obvious than those with SA and PA-T. In A-T, the roots of alfalfa and triticale were overlapped and intersected. In the meantime, N competition ability of cereal crops was stronger than that of legume crops. Therefore, triticale could compete more N and down-regulate the N concentration around the root of alfalfa^2^. Low N environment could stimulate the nodulation and N fixation of alfalfa. Moreover, root interaction of alfalfa and triticale is likely to cause root exudates to vary (e.g., isoflavone), thereby promoting the nodulation on the roots of alfalfa. Thus, the alfalfa with A-T could facilitate the nodulation and N fixation ability. The root interaction of alfalfa/triticale with NA-T was weaker than that with A-T, so the nodulation and N fixation ability with NA-T were lower than those with A-T. In SA and PA-T, there was no root interaction of alfalfa and triticale. Given this, the N concentration around root of alfalfa in SA and PA-T was higher than that in A-T and NA-T. In the meantime, no root interaction had lower concentration of root exudates around root of alfalfa than root interaction. In this regard, the nodulation and N fixation ability with no root interaction were lower than those with root interaction. As revealed by the mentioned results, the closer the crop roots interact, the better the nodulation form and N fixation ability of alfalfa in alfalfa/triticale cropping systems will be. Numerous studies have also reported that cereal crops neighboring with legume crops could noticeably promote N and N fixation ability of legumes when they were intercropped together. For instance, Santalla *et al*.^26^ suggested that cereal crops could facilitate the nodule formation and the growth of legume crops in cereal/legume intercropping. Faba bean intercropped with garlic obviously up-regulated the nodule numbers and nodules dry weight of faba bean^27^. Likewise, in the intercropping system of wheat and broad bean, the intercropped faba bean could up-regulate the nodule numbers, nodules dry weight and single nodule dry weight, suggesting that the intercropping could enhance the nodulation ability of faba bean^16^. The mentioned results may support that intercropping between alfalfa and triticale can enhance alfalfa’s ability to compete for nitrogen, and the nodulation and N fixation ability of alfalfa are derived from low N. In this regard, the closer the intercropped roots of alfalfa and triticale interact, the better the nodulation and N fixation in alfalfa will exhibit.

There exists a complicated relationship between root interaction and N concentration of the nodulation and N fixation ability of alfalfa. In a range of alfalfa/triticale cropping systems, the nodulation and N fixation ability under N_21_ are better than those under N_210_. On the whole, increasing N concentration input often leads to the reduction of the nodulation and N fixation ability of legumes^28^. According to our study, nodulation and N fixation ability of alfalfa were obviously inhibited with rising N concentration application. N assimilation in alfalfa might consume carbohydrates, thereby resulting in a reduction in the amount of carbohydrates supplied to the nodules. Thus, the inhibiting effect of N on nodulation and N fixation ability was involved in decreased carbohydrates, which were supplied to nodules. Hu *et al*.^17^ reported the inhibiting effect of higher N concentrations on nodulation and N fixation ability. The negative effect of mineral N on legume–rhizobia symbioses could stimulate the nodulation and N_2_ fixation in pea (*Pisum sativum* L.), soybean (*Glycine max* L.) and peanut (*Arachis hypogaea* L.)^29^. The authors further revealed that the lower N concentration could contribute to the nodulation and N fixation ability of alfalfa. Xia *et al*.^30^ also suggested that lower concentrations of N (<50 mg L^−1^) promoted the nodulation and N fixation ability of soybean, while higher concentrations of N (>50 mg L^−1^) significantly suppressed the nodulation and N fixation ability of soybean. Different N concentrations and root interaction had a range of effects on various parameters about nodulation and N fixation ability. N concentrations slightly impacted TNN and NA of alfalfa. TNN and NA at higher N levels were up-regulated by 1~22% compared with lower N level. However, ENN, ENN/TNN, PNW, SNW and PNF were significantly impacted as the different N concentrations by alfalfa. ENN, ENN/TNN, PNW, SNW and PNF at higher N levels were up-regulated by 20~240%, as compared with those at the lower N level. The results could be because different parameters regarding the nodulation and N fixation ability exhibit different N sensitivities. As compared with N21 and N210, nodulation and N fixation of alfalfa in different cropping systems displayed different amplifications. The diversifications between lower N level and higher N level of PNW, SNW and PNF in no root interaction systems were less obvious than those in no root interaction systems. It was because of the amplifications in root interaction and no root interaction of PNW, SNW and PNF were greater at lower N level, whereas the amplifications of PNW, SNW and PNF were less obvious at higher N level. Under the low N level, root interaction led to the down-regulated N concentration around alfalfa root. The low-down N concentration could inhibit the growth of plant cells, as well as the weight of nodules of alfalfa. Daimon *et al*.^31^ reported that N concentration greater than 14 mmol L^−1^ significantly suppressed the increase in nodules number and weight of peanut, whereas less than 14 mmol L^−1^ slightly impacted nodules number and weight of peanut^32^ Thus, alfalfa/triticale with A-T and NA-T under lower N made low-down N environment around root of alfalfa. On one hand, low-down N level and root exudates stimulated nodule development; on the other hand, low-down N level inhibited nodule cells from increasing. As a result, the diversifications of SNW by alfalfa between root interaction and no root interaction were less obvious at the low N level. PNW and PNF were primarily affected by SNW, so the differences of PNW and PNF between root interaction and no root interaction were less obvious at lower N level.

In this study, the differences on TNN of alfalfa with A-T and other cropping systems became greater at higher N level, yet their differences were enhanced and then became smaller at lower N level, so did ENN and NA of alfalfa with the progress of plant growth,. The competition and absorption ability for N were improved with the growth of alfalfa and triticale, while the N concentration around root of alfalfa became lower. When N concentrate around root of alfalfa reached a very low level, the production of nodules would be inhibited. For instance, Xia *et al*.^29^ reported soybean at the without N addition, the nodule numbers and nodules weight with increasing N concentration, and then decreased with rising N concentration. PNW and SNW with A-T and other cropping systems were less obviously amplified with the continuous growth of alfalfa. Root interaction and photosynthesis influence jointly affected nodule weight. On one hand, the root interaction was closer, and the SNW was larger. On the other hand, alfalfa exhibited weaker photosynthesis in A-T, NA-T and PA-T than that in SA. Weaker photosynthesis reduced carbohydrate content of alfalfa, thereby affecting the weight of nodule. Moreover, nodules weight was affected by root interaction and photosynthesis. Over the growth period, the photosynthesis influence was greater than root interaction, so the differences between different cropping systems were less obvious. The effect of alfalfa on nodulation and N fixation ability might be regulated and impacted by the aboveground and underground parts of the plant^29^. In the meantime, N-induced suppression of nodulation and N fixation ability was regulated by the underground and aboveground portion of the alfalfa. Besides, there have different contributions of underground and aboveground portions to different parameters regarding the nodulation and N fixation ability. The result complies with other studies, finding different contributions of root interaction and photosynthesis on nodulation and N fixation ability.

### Root interaction facilitates dry weight and N accumulation

PDW of alfalfa with different cropping systems displayed slight differences. However, PDW of triticale showed obvious differences. PDW of triticale with A-T was higher than that with NA-P, and that with NA-P was higher than that with PA-T and ST. It was therefore suggested that triticale was the dominant specie in alfalfa/triticale cropping system and exhibited resource competitiveness, covering the competition for light energy and N. Likewise, in legume/non-legume intercropping system, the non-legume plants were usually dominated, and such advantage became more obvious as N levels rose^33^. In the present study, TDW of alfalfa with A-T was higher than those with SA and ST at the initial flowering stage, suggesting that intercropping could up-regulate biomass per unit area. In contrast, interspecific competition commonly existed in cereal/cereal intercropping, when one species reduced the growth and/or depressed the yield of another species^34^. However, Hua *et al*.^35^ reported that compared with corresponding monoculture crops, intercropped pea increased the N_2_ fixation by 34%, and the grain yield of intercropped pea and maize was up-regulated by 37% and 29%, respectively. PNA of alfalfa in no root interaction was higher than that in root interaction at the flowering stage. The ability of alfalfa to fix N was lower than that of triticale to fix N^35^. However, PNA of triticale exhibited an opposite performance. PNA of triticale with root interaction was higher than that with no root interaction at three growing stages. Xiao *et al*.^36^ compared sola barley; they reported that the N content in the shoot and root of intercropped barley increased by 5.8~35.8%, and N accumulation in the aboveground was up-regulated by 7~32.1%. In this study, TNA with A-T was higher than those of other cropping systems of alfalfa and triticale at the initial flowering stage under N_210_. Nevertheless, under N_21_, TNA with SA was higher than that with A-T, and TNA with ST was the lowest. The mentioned results revealed that TNA could exhibit its advantages at higher N level; while at lower N level, intercropping of N accumulation was second to sole alfalfa. These positive results could be attributed to the interspecific promotion (e.g., making complimentary use of atmospheric N_2_ resources, up-regulating N utilization efficiency, and cooperating rooting system to enhance abiotic and biological stress)^18^.

In the present study, we found that the parameters about nodulation and N fixation ability were significantly positively correlated. This was because the nodulation and N fixation ability were positively correlated with root interaction and negatively correlated with N concentration. Accordingly, when the experimental conditions are not perfect, the NA and PNF of alfalfa can be roughly speculated by ascertaining alfalfa nodule number and nodule weight. According to Agegnehu *et al*.^3^, the direct determination of N fixation is of high cost; the number of leguminous nodules or nodule weight conditions was employed to assess the N fixation ability of leguminous plants since leguminous nodulation is significantly correlated with N fixation. In this study, parameters of nodulation and N fixation ability except for nodule weight are significantly positively corrected with TNA and TDW, revealing that the nodule weight cannot act as a direct parameter to assess the intercropping advantage. Accordingly, alfalfa production can be promoted by increasing nodule numbers and N fixation ability in a certain range, and the promotion of root interaction can lead to the increase in the potential for simulated alfalfa/triticale intercropping.

## Conclusions

In different cropping systems, the closer roots interaction, the better the nodulation form and N fixation ability of alfalfa will be, and the higher the biomass and N accumulation of all plants in pots will be. Interspecific facilitation in alfalfa/triticale intercropping system resulted in a greater yield and N accumulation; it also ultimately enhanced nodulation and N fixation ability, which can be applied in sustainable systems to avoid N loss to the environment and enhance N use efficiency.

## Materials and Methods

### Experiment design and plant management

The experiment was conducted with sand culture in plastic pots (32 cm diameter, 20 cm height) in a growth chamber at Gansu Agricultural University (GAU), Lanzhou, China. In the growth chamber, light was at 28 °C/14 h and darkness was at 20 °C/10 h, light intensity was 260~350 mol m^−2^ s^−1^, and relative humidity was 60~70%. It consisted of two N treatments and five cropping systems. The two N treatments were (1) the low N level, 21 mg N L^−1^ as mixed N source of Ca(NO_3_)_2_ and (NH_4_)_2_SO_4_ (N21), (2) the medium N level (appropriate nitrogen levels of alfalfa), 210 mg N L^−1^ as mixed N source of Ca(NO_3_)_2_ and (NH_4_)_2_SO_4_ (N210). Hoagland-Arnon solution as the basic nutrient solution was used in the N21 and N210 nutrient solutions, and the ration of NO_3_^−^ -N: NH_4_^+^ -N was 1:1. The five cropping systems were (1) alfalfa (cv. LW6010, provided by company of Mammoth Seed) as a monoculture (SA), (2) triticale (cv. Zhongsi 1048, provided by Hebei Academy of Agriculture and Forestry Sciences, China) as a monoculture (ST), (3) simulated alfalfa/triticale intercropping with no barrier (A-T), (4) simulated alfalfa/triticale intercropping with nylon mesh barrier (NA-T), (5) and simulated alfalfa/triticale intercropping with plastic barrier (PA-T). Plastic pots (0.47 m diameter) were cut in the middle, separated into two compartments with nylon mesh (diameter of 0.2 mm) or plastic placed in the middle, and then reconstructed^37^. The alfalfa and triticale phenotypes can be found as “Supplementary Material”. The A-T planting pattern had no artificial physical barrier between alfalfa and triticale, allowing water and nutrients to exchange and possible root interaction between alfalfa and triticale. NA-T planting pattern obstructed overlapping of alfalfa roots and triticale roots but allowed water and nutrients to exchange through the nylon meshes. The PM-P planting pattern prevented water and nutrients exchanged between alfalfa and triticale with no overlapping of alfalfa roots and triticale roots. There were 3 replications in each treatment.

The seeds of alfalfa and triticale were chosen and disinfected, and then were planted in separated one side of plastic pots filled with sand. When the alfalfa and triticale grew to 3 cm in height, healthy seedlings were kept in each plastic pot with the rest removed by hand. SA and ST kept 20 seedlings, A-T, NA-T and PA-T of alfalfa and triticale kept 10 seedlings, respectively. Seven days after emergence of alfalfa, two levels of N nutrient solution (1000 mL per pot) were added to the pots, and then rhizobium (*Sinorhizobium meliloti*, 12531, provided by College of Pratacultural Science, GAU) liquid was inoculated (25 mL per pot, OD600 between 0.63 to 0.64). Sand was rinsed with distilled water and nutrient solution was replaced once a week. Distilled water (500 mL) was slowly added in each pot (prevent salt ion accumulation) in each time. Then pots with plants were rinsed with distilled water for 12 h, and then added nutrient solution (1000 mL per pot). Distilled water was supplemented every day to the location of the nutrient solution application for the first time.

Sowing date for alfalfa was 12 March 2017, and harvest dates were 8 June 2017 (branching stage), 24 June 2017 (budding stage) and 11 July 2017 (initial flowering). Sowing date for triticale was 20 April 2017, and harvest dates (jointing stage, booting stage and heading period) were the same as alfalfa.

### Plant sampling and nodule collection

Both alfalfa and triticale were sampled at three growth stages: alfalfa branching stage (50% of alfalfa plants occurred lateral branching in the pots), alfalfa budding stage (50% of alfalfa plants flower budded in the pots), and alfalfa initial flowering stage (20% of alfalfa plants flowered in the pots). During the sampling stage, 10 individual alfalfa and triticale plants were selected from each pot for nodulation, nitrogenase activity, dry matter and nitrogen accumulation tests.

### Nodulation

Roots were rinsed with distilled water, and absorbed residual water with absorbent paper, then the nodules were removed quickly. All fresh nodules were detached from the roots to collect, counted and weighted. Nodulation, including total nodule number (TNN), effective nodule number (ENN), the ratio of effective nodule number/total nodule number (ENN/TNN), fresh nodule weight per plant (PNW), single fresh nodule weight (SNW) of alfalfa were measured. At the time of sampling, pink nodules representing their high efficiency in N fixation were regarded as ‘effective nodules’ while immature or aged nodules in yellow or grayish brown were regarded as ‘non-effective nodules’, and the ratio of ENN to TNN was calculated accordingly. The nodulation phenotypes of alfalfa can be found as “Supplementary Material”.

### Nitrogenase activity

The nitrogenase activity (NA) was measured by the acetylene reduction assay^38^. 0.2 g fresh nodules were weighted and put into a 7 ml glass bottle sealed with a rubber stopper. 10% volume of air was removed and replaced by equal volume of acetylene. After 30 min at room temperature, duplicate 25 μL gas samples were removed and analyzed by gas chromatography for the peak of ethylene and acetylene. The standard curve of ethylene was determined and measured under standard conditions using standard ethylene to calculate the nitrogenase activity of the nodule sample. The instrument was the GC-7890F gas chromatograph with column temperature of 180 °C, sampler of 150 °C, and FID detector of 170 °C. Gas pressure: N_2_ is 0.3 mPa, H_2_ is 0.08 mPa, and air is 0.15 mPa. C_2_H_4_ level (μmol g^−1^ h^−1^) = hx (sample peak area) × C (standard C_2_H_4_ level, μmol/mL)/hs (standard C_2_H_4_ peak area) × 24.9 × t (C_2_H_2_ reaction time, h) × m (tumor weight, g).

### Plant dry matter weight

During the sampling period, the whole 10 plants of alfalfa and triticale were collected from each pot for determining plant dry matter weight. Whole plant dry weight (PDW, mg plant^−1^) was determining by putting fresh samples in 105 °C oven for 15 min and then in 60∼70 °C oven till a constant weight. Dry weight per pot (TDW) was the dry matter weight of whole plants in each pot (mg pot^−1^) = (PDW of alfalfa + PDW of triticale) × 10.

### Plant N accumulate

Plant nitrogen content (PNC) was determined by Kjeldahl procedure after digestion in a mixture of concentrated H_2_SO_4_ and H_2_O_2_^39^. Smashed sample was digested in the Kjeldahl digestion flask by boiling with H_2_SO_4_-H_2_O_2_ until the mixture became clear. The digested liquid was filtered and volume. Ammonia was steam distilled from the digest to which NaOH solution was added. The distillate was collected in a conical flask containing HCl and red methyl indicator. The ammonia that was distilled into the receiving conical flask reacted with the acid and the excess acid in the flask was estimated by back titration against NaOH with color change from red to yellow (end point). Determinations were made on all reagents alone (blank determinations). N (%) was calculated as [(ml standard acid × N of acid) − (ml blank × N of base)] × (ml std base × N of base) × 1.4007/Weight of sample in grams × 100%. Plant N accumulation (PNA, mg per plant) = PNC × PDW. Nitrogen accumulate per pot (TNA) of alfalfa and triticale (mg per pot) = (PNA of alfalfa + PNA of triticale) × 10

### Statistical analysis

Data were analyzed using Statistical Analysis Software (SPSS software, 17.0, SPSS Institute Ltd, USA) with the standard split-plot design analysis method to test for significance of treatments, and means were compared by least significance difference (LSD). N treatment and cropping system were considered as fixed effects and replication as random effects. Correlation analysis of different parameters adopted linear correlation analysis. All significances were declared at the probability level of 0.05.

## Supplementary information

Supplementary information
