# Supplementary material for: Effect of root interaction on nodulation and nitrogen fixation ability of alfalfa in the simulated alfalfa/triticale intercropping in pots
Source: Sci Rep. 2020 Mar 6;10:4269. doi: 10.1038/s41598-020-61234-5 (PMC7060277; doi:10.1038/s41598-020-61234-5)
Supplement: Supplementary file 1 — Supplementary information [file 41598_2020_61234_MOESM1_ESM.pdf]

Effect of root interaction on nodulation and nitrogen fixation ability of alfalfa in the simulated alfalfa/triticale intercropping in pots

Yajiao Zhao, Xiaojing Liu * , Changchun Tong, Yong Wu

Alfalfa, cv. LW6010, provided by company of Mammoth Seed. The fall dormancy of LW6010 is the 6th level. It has the strong salt and alkali resistance and disease and insect resistance. Its plant has strong upright character, and its natural plant height reaches 60 ~ 70 cm. LW6010 has many tillers, thick stalks, large and thick leaves, high ratio of leave to stem, and often appears leafy phenomenon. In flied experiment, the fresh grass yield of LW6010 was 55.3 ~ 57.6 t hm^{-2}, and the dry yield of it was 11.0 ~ 13.0 t hm^{-2}. At the flowering stage, its crude protein content was 18% ~ 19%; ADF was 32% - 35%; and NDF was 43% ~ 48%; crude fat was 2.6% ~ 2.7%.

Triticale, cv. Zhongsi 1048, provided by Hebei Academy of Agriculture and Forestry Sciences, China. Zhongsi 1048 is hexaploid titriticale with strong winter resistance, late maturity, disease resistance, drought resistance, cold resistance and lodging resistance. Its plant height is 150 ~ 180 cm. The characteristics of Zhongsi 1048 are many tillers, thick stems, alternate leaves, large leaves, thick green leaves with wax, high ratio of leaves to stems, luxuriant stems and leaves, etc. In flied experiment, the fresh grass yield of LW6010 was 42.0~49.5 t hm^{-2}, and the dry yield of it was 10.5~16.5 t hm^{-2}. At the flowering stage, its crude protein content was 15% ~ 16%; ADF was 36% ~ 39%; and NDF was 46% ~ 50%; crude fat was 7.8% ~ 8.4%.

The root nodule size of LW6010 is roughly the same as that of rice grain. The shape of the nodule was three petal palmar. The nodules are yellow when they are immature, pale pink when they are mature, and grayish brown when they are old. At the same time, the nodules of LW6010 are indeterminate. Indeterminate nodules are cylindrical, arise from root pericycle and inner cortical cells, and contain a persistent terminal meristem. Indeterminate nodules grow primarily bycell division at the terminal meristem.
